# Noise and Quality of Life

**DOI:** 10.3390/ijerph7103730

**Published:** 2010-10-19

**Authors:** Michael D. Seidman, Robert T. Standring

**Affiliations:** Henry Ford Health System, Director Division Otologic/Neurotologic Surgery, Medical Director Center for Integrative Medicine and for Wellness, 2799 W Grand Blvd, Detroit, MI 48202, USA; E-Mail: rstandr1@hfhs.org (R.T.S.)

**Keywords:** noise, oxidative stress, quality of life, noise and non-auditory health

## Abstract

Noise is defined as an unwanted sound or a combination of sounds that has adverse effects on health. These effects can manifest in the form of physiologic damage or psychological harm through a variety of mechanisms. Chronic noise exposure can cause permanent threshold shifts and loss of hearing in specific frequency ranges. Noise induced hearing loss (NIHL) is thought to be one of the major causes of preventable hearing loss. Approximately 10 million adults and 5.2 million children in the US are already suffering from irreversible noise induced hearing impairment and thirty million more are exposed to dangerous levels of noise each day. The mechanisms of NIHL have yet to be fully identified, but many studies have enhanced our understanding of this process. The role of oxidative stress in NIHL has been extensively studied. There is compelling data to suggest that this damage may be mitigated through the implementation of several strategies including anti-oxidant, anti-ICAM 1 Ab, and anti JNK intervention. The psychological effects of noise are usually not well characterized and often ignored. However, their effect can be equally devastating and may include hypertension, tachycardia, increased cortisol release and increased physiologic stress. Collectively, these effects can have severe adverse consequences on daily living and globally on economic production. This article will review the physiologic and psychologic consequences of noise and its effect on quality of life.

## 1. Introduction

Noise is typically defined as an unwanted sound or a combination of sounds that may adversely affect people. Noise can manifest in the form of physiologic damage or psychological harm. The mechanism of physiological damage from noise has yet to be fully understood, but research has demonstrated a multitude of factors including increased oxidative stress, vascular changes, and mechanical trauma may be responsible, to name a few. Psychological harm from noise exposure may manifest as increased physiologic stress response, adverse social consequences, sleep disturbance, and detrimental economic effects [1,2]. Noise induced hearing loss (NIHL) has global implications, with 10 million adults and, 5.2 million children in the US, and 250 million people worldwide having a NIHL greater than 25 dB, a clinically significant hearing loss [3,4]. Additionally, occupational noise accounts for 16% of the disabling hearing loss in adults worldwide resulting in decreased economic production [5]. Currently, the armed forces are facing severe disabilities secondary to noise. It has been shown that the top two disabilities now facing the American military are hearing loss and tinnitus. The US government is predicted to spend nearly 1.6 billion dollars this year in order to rehabilitate the men and women traumatized from the effects of noise (TATRC 2010). The purpose of this article is to review some of the recent literature on the physiological and psychological effects from noise and its relationship with quality of life.

## 2. Mechanisms of Noise Induced Hearing Loss

Noise induced hearing loss is generally defined as hearing loss that develops slowly over a long period of time (several years) as the result of exposure to continuous or intermittent loud noise [6]. This results in bilateral, sensorineural hearing loss, often with a pathognomonic notch of decreased hearing on an audiogram at 4,000 Hz. Continuous exposure to sounds greater than 85 dB for 8 hours has been shown to cause NIHL [7,8]. Furthermore, when the exposure is constant, there is increased damage compared to intermittent noise exposure of similar intensity [6,8]. The effects of noise damage are seen more in males, which is possibly due to a larger percentage of males in specific work environments [5].

The role of genetics has been a recent area of interest in uncovering the causative mechanisms of NIHL. There is anecdotal evidence suggesting a possible genetic role. It has been demonstrated that certain patients are more susceptible to permanent threshold shifts (PTS) based on auditory brainstem response (ABR) threshold measurements when subjected to a similar noise exposure [9,10]. To date, any loci of susceptibility to NIHL have not been identified in humans, but many mouse models have been shown to promote NIHL [11]. Chen *et al.* [12] found that mice deficient in a gene Xp21.2, mapped in the dystrophin gene locus, were more vulnerable to NIHL compared to control mice. Schick *et al*. [13] demonstrated that there was an increase in noise sensitivity at lower frequencies in vasodilator-stimulated phosphoprotein −/− mice. Fairfield *et al*. [14] investigated the relationship of NIHL and the role of heat shock proteins (HSPs), molecules that can enhance cell survival in response to stress, and heat shock factor 1 (HSF1), the major transcription factor that regulates HSPs expression in stress, in mice. They found that HSF1 −/− mice were found to have more hearing loss than normal mice given the same noise exposure. Morita *et al*. [15] looked at the gene Ahl3, located in the 14-Mb region on the mouse chromosome 17 and its role in NIHL. They found a resistance to NIHL after prolonged noise exposure in mice when the wild type Ahl3 allele was present, demonstrating a genetic variation in susceptibility to NIHL.

Hearing loss from otologic trauma, which is defined as a sudden change in hearing due to exposure of a sudden burst of sound, may be due to a different mechanism of action than NIHL, and should be thought of as a separate entity. Acoustic trauma can cause mechanical disruption of the cochlea and may result in permanent hearing loss. In 1944, Lurie *et al*. [16] described graduated degrees of acoustic trauma in guinea pigs beginning with stripping of mesothelial cells under the basilar membrane, progressing to disruption of external hair cells and eventually, separation of the Organ of Corti from the basilar membrane as the time of exposure increased. Noise induced hearing loss, on the other hand, is thought to be due to various other mechanisms. One leading theory that will be focused on in this article is the role of oxidative stress in chronic noise exposure.

### Oxidative Stress

Pathology arising from increased metabolic activity in the inner ear was originally proposed by Lim and Melnick (1971) [17] and gained popularity with work by Yamane in 1995 [18,19]. Presently, there is an abundance of literature exploring the role of oxidative stress in NIHL [20,21]. Exposure to noise has been demonstrated to cause an initial increase in cochlear blood flow. Within a short period of time, there is an abrupt decrease in cochlear circulation seen by an aggregation of RBCs, capillary vasoconstriction, and stasis [22]. This intense metabolic activity and decreased cochlear blood flow from noise exposure alters cellular redox states and drives the formation of free radicals [23]. These free radicals may also be generated by a variety of mechanisms. They may form *invivo* as a byproduct of mitochondrial respiration, called Reactive Oxygen Species (ROS), arise from environmental contaminants, or can be produced from ionizing and ultraviolet radiation [21]. ROS include the superoxide ion (O_2_^−^), hydrogen peroxide (H_2_O_2_), and the hydroxyl radical (OH^−^), hypochlorite (OCl^−^), and nitric oxide (NO^−^) to name a few. In excess, ROS can damage cellular DNA, proteins, and lipids, as well as up regulate apoptotic pathways causing cell death and irreparable damage to eloquent hearing structures [24].

Research on oxidative stress has spawned subsequent attention toward the protective role of antioxidants in NIHL. The senior author has published multiple studies on the role of anti-oxidants in protection against NIHL. One of these studies evaluated the effects of resveratrol on NIHL. Resveratrol is a substance found in the skin of grapes and over 70 other fruits and plants. It has many beneficial biological properties that include inhibiting lipid peroxidation, free-radical scavenging, anti-inflammatory activity, inhibiting platelet aggregation, vaso-relaxing activity, anti-cancer properties, and cardio-protection to name a few [25–27]. In this 2003 study by Seidman *et al*., Fischer rats were randomized into a treatment and control group. The treatment group was pretreated for 3 weeks with resveratrol solution (430/ug/kg/day) prior to a 24-hour exposure to a 105 dB noise at 4,500 to 9,000 Hz. The treatment group were then given four weeks of post-stimulus treatment while the control group received only saline. The results from this study demonstrated a significant reduction of auditory threshold shifts in the treatment group compared to the controls. There was also a decrease in the loss of hair cells in the Organ of Corti in the treatment group compared to the control group. These findings demonstrated that the resveratrol anti-oxidant properties can be protective against NIHL, strengthening the hypothesis that noise damage is due to oxidative stress [28].

Another study by the senior author in 2009 [29] investigated the effects of anti-intracellular adhesion molecule-1 antibody (Anti-ICAM-1 Ab) on NIHL. ICAM-1 leads to neutrophil endothelial cell adhesion [30] which can subsequently lead to increased circulating levels of cytokines, leukotrienes, thromboxanes, platelet activating factor, complement components, elastases, and other enzymes, as well as additional formation of ROS [31]. In this study, Fischer rats were randomized into a control group with saline and a treatment group where they received intravenous Anti-ICAM-1 Ab prior to a 72-hour period of 107 dB SPL noise. The groups then received another dose of Anti-ICAM-1 Ab or saline 24 hours after noise exposure. The rats treated with Anti-ICAM-1 Ab showed attenuated temporary threshold shifts (TTS) compared to the control group. This study demonstrated that by using Anti-ICAM-1 Ab to block the cascade of ROS, rats were protected against NIHL, strengthening the evidence of the oxidative stress hypothesis [29].

There have been numerous studies on the role of oxidative stress in hearing loss and the ability of various anti-oxidants to attenuate the physiological damage. Coleman *et al*. [32] found that N-Acetylcysteine and acetyl-L-carnitine both significantly reduced permanent threshold shifts and hair cell loss in mice compared to saline-treated controls when exposed to a consistent noise. Samson *et al*. [33] found that the anti-oxidant D-methionine reversed the change in lipid peroxidation and significantly reduced permanent threshold shifts on day 14 from 15 dB to 5 dB at the 4 kHz range in mice. There are many other studies that have investigated the role of anti-oxidants in NIHL and we suggest the reader to look at the cited review articles for more information [21,34,35].

Another hypothesis looked at the role of glutamate in NIHL. Noise exposure is known to cause excessive excitatory synaptic activation of glutamate receptors and leads to glutamate excitotoxicity. This process ultimately results in neural swelling through water and calcium influx, which induces necrotic and apoptotic cell death in the spiral ganglion. This results in permanent mechanical changes and subsequent hearing loss [20,36]. Many of these theories may contribute to the mechanism of NIHL.

## 3. Physiological Response to Noise

Noise can have a more global effect on human physiology and act upon multiple non-auditory systems such as cardiovascular, neuroendocrine, and psychological [37]. Quantifying the non-auditory effects of noise may be difficult due to lack of strong scientific evidence lack statistical evidence, as there are often plausible alternative explanations for the results. These studies often have confounding variables, especially selection bias.

There have been multiple studies that have investigated the relationship between noise, blood pressure, and myocardial damage. A comprehensive meta-analysis by van Kempen *et al*. [38] looked at 43 epidemiological studies looking at the correlation between noise exposure and elevated blood pressure and ischemic heart disease during the time period of 1970 and 1999. They found that there was a significant association between occupational and air-traffic noise exposure and hypertension. The evidence of noise exposure causing ischemic heart disease was inconclusive as there was no definitive relationship.

Another study investigated the relationship of noise and blood pressure and a stress response included a quality of life survey to noise exposed subjects. Evans *et al*. [2] looked at children that lived in a noisy “airport” community and a quiet community. They measured systolic and diastolic blood pressure, urine epinephrine, norepinephrine, and cortisol levels, as well as obtained results from the KINDL quality of life questionnaire. They found that there was an increase in blood pressure in the noisy communities. There was also a significant increase in urine epinephrine and norepinephrine levels, but not a significant change in cortisol levels. The KINDL questionnaire is an assessment of quality of life in children and has been proven to produce reliable results [39–41]. This questionnaire was used in this study and found that the quality of life in the children living in the noisy community declined significantly over the 18 months of the study [2].

### Sleep Disturbance and Cognitive Impairment

Noise can cause disturbances in sleep and subsequent deleterious health effects and perceived decrease in quality of life [42]. Noise can cause immediate or secondary extra-auditory effects. The number and duration of nighttime awakenings can quantify the immediate effect of noise on sleep. Nocturnal awakenings usually occur with noise levels greater than 55 dB [42]. The time that it takes to fall asleep can increase up to 20 minutes with peak noise levels of 45 dB [43]. After approximately 5 hours of sleep the threshold of awakening lightens and noise can cause frequent early morning awakenings [42]. The secondary effects of noise on sleep are a subjective feeling of decreased quality of sleep, tachycardia, increase in stress hormones (as discussed previously), and increased cognitive impairment [44]. The perceived quality of sleep can be quantified by using any number of objective questionnaires, some of which include the SF-36, the Nottingham Health Profile, or the Functional Outcomes of Sleep Questionnaire to name a few [45–48]. These effects of noise on the sleep process may also contribute to the impairment of cognitive tasks and overall performance [44].

Noise may cause cognitive impairment from a variety of mechanisms. Earlier studies have demonstrated that children in noisy environments have decreased attention on tasks and have lower performance on cognitive assignments compared to children in quiet environments [49–52]. A more recent study by Ljung *et al*. [53] found that traffic noise significantly impaired reading ability and comprehension as well as basic mathematic performance in children. Previous hypotheses suggested that the cognitive impairment from noise was due to cognitive coping where children “tune out” excessive stimulation and have a generalized poor attention [52,54]. Another hypothesis was that noise caused a high level of arousal and resulted in an inability to concentrate [55,56]. These psychological and physiological non-auditory effects of noise result in detrimental health consequences and a decreased quality of life.

## 4. Conclusions

This is just some of the evidence to highlight noise as an unwanted environmental pollutant that has global implications. In our industrialized society, a significant population is exposed to noise on a daily basis with its resultant health effects, and subsequent substantial economic burden. There has been a plethora of research on the mechanism of NIHL. The role of oxidative stress in NIHL has been extensively investigated and remains a probable cause of NIHL. This results in significant deterioration in quality of life in that it disrupts sleep, causes cognitive impairment, and has many non-auditory deleterious health effects.
